# Development of a computer-tailored nutrition and physical activity intervention for lower-educated women of Dutch, Turkish and Moroccan origin using content matching and ethnic identity tailoring

**DOI:** 10.1186/s12889-016-3596-6

**Published:** 2016-09-02

**Authors:** Kristina Romeike, Lilian Lechner, Hein de Vries, Anke Oenema

**Affiliations:** 1 School for Public Health and Primary Care (Caphri), Maastricht University, PO Box 616, 6200 MD Maastricht, The Netherlands; 2 Department of Health Education and Health Promotion, Maastricht University, PO Box 616, 6200 MD Maastricht, The Netherlands; 3 Faculty of Psychology and Educational Science, Open University of the Netherlands, PO Box 2960, 6401 DL Heerlen, The Netherlands

**Keywords:** Nutrition, Physical activity, Computer tailoring, Print-delivered intervention, Ethnic identity tailoring, Turkish, Moroccan, Dutch, Evidence-based, Theory-based

## Abstract

**Background:**

Unhealthy dietary and physical activity (PA) patterns are highly prevalent in most Western countries, especially among lower-educated and ethnic minority groups. Therefore, interventions to promote healthy eating and physical activity that can reach large numbers of lower-educated people are needed. When developing interventions, the ethnic diversity of the lower-educated population may be taken into account to make intervention material more appealing to the target group. This article describes the development and evaluation of two computer-tailored nutrition and physical activity interventions for lower-educated Dutch, Turkish and Moroccan women. One version is tailored to sociocognitive variables (traditional tailoring), while the other is additionally tailored to ethnic identity (EI-tailoring).

**Method:**

Using intervention mapping, two evidence- and theory-based interventions were developed. In the traditional tailoring intervention, messages are tailored to health behavior, awareness of own behavior, attitude and self-efficacy. The behavior change techniques used to address these factors are: descriptive and evaluative feedback, arguments, modeling, goal setting, planning, barrier identification and advice on how to deal with barriers, stimulating resistance to social pressure, mobilization of social support (nontailored), active learning (nontailored) and iterative feedback. In the EI-tailoring intervention, the material is additionally tailored to ethnic identity (EI). This means that recipients who feel strongly attached to their ethnic background receive different intervention material than recipients with a weak attachment to their background. This includes, for instance, the use of more traditional colors, role models that match with their origin and advice messages that refer to their ethnicity of origin.

**Discussion:**

Developing an intervention that matches the needs of this specific target population was challenging due to the little evidence regarding the determinants of their health behavior, as well as the behavioral change techniques that have not been tested among Turkish and Moroccan women in the Netherlands before. Based on previous research among this and other target populations we hypothesize, however, that the determinants and strategies we use will be suitable. A randomized controlled trial will show whether the interventions are effective among our specific target group and whether EI-tailoring is beneficial.

**Trial registration:**

Dutch Trial Registry NTR4506, registration date: 1^st^ may 2014

## Background

Unhealthy dietary and physical activity (PA) patterns are highly prevalent in most Western countries [1–3] and even more so among lower-educated and ethnic minority groups [4–10]. More specifically, low fruit and vegetable intake, high consumption of energy-dense products and high percentages of people not meeting the PA norms have been reported for Western populations [2, 10–13]. Unhealthy dietary and PA behavior have been associated with negative health outcomes, such as type 2 diabetes, cancer and cardiovascular diseases [14, 15]. Lower-educated and ethnic minority groups are at higher risk of these health problems [4, 16–18]. It is, therefore, important to improve dietary and PA behaviors among lower-educated and ethnic minority groups in particular. Moreover, since the problem occurs on such a large scale, interventions are needed that can reach large numbers of people.

In the Netherlands, only 3–14 % of the general population meet the norm for vegetable consumption, and only 3–26 % consume a sufficient amount of fruit [19]. The Health Council of the Netherlands recommends eating at least 200 grams of vegetables and 200 grams of fruit every day [20]. The Netherlands Nutrition Center (Stichting Voedingscentrum Nederland) even recommends to eat 250 grams of vegetables or more [21]. The results of the most recent Dutch National Food Consumption Survey [19] showed that 25 % of adult women consume 2200 kilo-calories per day or more. In-between meal snacks, such as sugar, confectionery and cake, accounted for as much as 21 % of the daily energy intake [19]. Studies among populations with a low socioeconomic status and a lower education have shown that a low fruit and vegetable consumption is even more common in these groups [4–6], and that their snack consumption is higher [22].

In the Netherlands there is a variety of ethnic minorities, with Turks and Moroccans being among the largest groups [23]. Studies among ethnic minority groups in general [9], and Turkish and Moroccan groups in particular [24, 25], have shown that high percentages of the adults do not meet the norm for fruit and vegetable consumption. The limited available evidence on the snack consumption of Turkish and Moroccan groups in the Netherlands shows that Turkish and Moroccan women snack a lot in between meals [25, 26]. In-between meal snacks comprise 28 % of the total daily energy intake of Turkish participants, and 22 % of that of the Moroccan participants [25].

The percentage of people in the general Dutch population meeting the norm for moderate PA has been relatively constant for a number of years at around 59 %, with working, transport and household activities being the main contributors [7]. It is recommended to be moderately physically active (e.g. cycling, walking) for 30 minutes a day, on at least five days per week [7]. Lower-educated people are even less physically active than higher educated populations [27, 28]. Furthermore, research shows that only 25 % of the Moroccan and 34 % of the Turkish people living in the Netherlands meet the norm for moderate PA [7]. Recent research shows that among Turkish and Moroccan women, only 39 % meet the Dutch norm for PA [29].

To improve the dietary intake and PA levels of lower-educated and ethnic minority groups a project was initiated aimed at developing and evaluating an intervention for these specific groups. We chose to include Turkish and Moroccan people in our target population (besides people of Dutch origin), since these are among the largest immigrant groups in the Netherlands. We furthermore chose to focus on women, as a needs assessment among Dutch, Turkish and Moroccan men and women revealed that it is mainly the women that prepare the meals for the family [30]. On consulting other studies we found that the Turkish and Moroccan target populations consider cooking to be the task of women [26, 31]. As a result, we felt that focusing on women may be the most efficient approach for our intervention. Therefore, the intervention described in this paper focuses on lower-educated women of Dutch, Turkish and Moroccan origin. We aimed to develop an intervention that can reach large numbers of people, as interventions for this specific target population that have a high reach and can be implemented on a large scale are rare in the Netherlands.

An effective intervention technique that can reach large numbers of people with personally relevant information is computer tailoring [32]. In computer-tailored interventions individualized feedback and advice can be provided, which is thought to be necessary and very effective in changing complex health-related behaviors such as diet and PA. Relevant information on individual characteristics, behaviors and beliefs is first assessed and subsequently feedback and advice are provided that match the personal circumstances, behaviors and beliefs of each individual [33]. This technique has been shown to be effective among many target groups and a variety of health behaviors, including dietary and PA behavior [34–37]. There is also evidence that computer tailoring interventions can be effective for lower-educated people [38–40].

Hawkins et al. [41] argue that messages are likely to be perceived as more relevant when presented in a context that is significant to the individual, such as the cultural or demographic context, identity, ethnicity or personal interest. Consequently, by taking relevant contextual factors into account, tailoring messages may further increase the probability that the information provided is attended to and processed by groups with a specific contextual background. In other words, contextualizing intervention materials may enhance the chance of processing information centrally, as suggested by Hawkins et al. [41].

Improving attention may, in particular, be important for lower-educated and ethnic minority groups. We applied ethnic identity tailoring (EI-tailoring) to contextualize our intervention material to make it more attractive to the various sub-groups within our target population. Ethnic identity (EI) is the degree to which a person identifies with a particular ethnic group [42]. U.S. studies among African American populations have demonstrated that taking the EI or the cultural background into account when promoting a healthy lifestyle can improve the effectiveness and/or the attractiveness of health-promoting interventions [42, 43]. We used EI as a concept to contextualize our intervention materials.

The aim of this paper is to describe the systematic development of the two versions of a computer-tailored intervention aimed at promoting a healthy diet and a sufficient amount of PA among lower-educated Dutch, Turkish and Moroccan adult women living in the Netherlands.

## Method

We used the intervention mapping (IM) protocol to develop a computer-tailored intervention, as it helps to structurally develop a theory- and evidence-based intervention. IM follows six steps that are described by Bartholomew et al. [44]: 1) conduct a needs assessment, 2) create matrices of change objectives, 3) select theory-based intervention methods and practical applications, 4) develop an intervention program, 5) plan for the adoption, implementation and sustainability of the program and 6) plan the evaluation of the intervention.

### Creating the base of the intervention

Developing an intervention starts with a thorough assessment of the needs within a target group. This needs assessment results in a definition of the behaviors that should be addressed in order to improve the health of the target population, as well as a definition of specific sub-behaviors related to the behavior and their determinants. These (sub-)behaviors and their determinants create the base for an intervention. The needs assessment, which is briefly summarized in the introduction section, led us to focus on four behaviors that we aim to address with our intervention: fruit consumption, vegetable consumption, snack consumption and physical activity. Therefore, the aim of the interventions described in this paper is to improve these behaviors among the target group.

The follow-up brochure contains advice about both be-Self-regulation theories were used to formulate *performance objectives* indicating which specific sub-behaviors people should master to be able to successfully change the target behaviors. Self-regulation theories [45] state that behavior change emerges through a process of two phases. The process begins with a *pre-intentional*, *motivational phase* that includes problem identification, goal selection and goal setting. The next step involves the *post-intentional*, *volitional phase*, encompassing the translation of the goal into action by making a plan and considering solutions for how to deal with difficult situations. Additionally, the behavior must be evaluated and monitored in this phase to facilitate maintenance of the change over time [45]. The performance objectives were formulated in line with these self-regulation phases (see Table 1).

**Table 1:** Performance objectives, represented by each phase of the self-regulation process

Motivational phase
After exposure to the intervention…
PO 1: Individuals from the target group decide to eat more fruit, more vegetables, or fewer high-energy snacks, or to engage in more PA.
PO 2: Individuals set a specific, relevant, appropriate and feasible goal to eat more fruit, more vegetable, or fewer high-energy snacks, or to engage in more PA.
Volitional phase
After exposure to the intervention…
PO 3: Individuals think of strategies that may help them eat more fruit, more vegetables, or fewer high-energy snacks, or to engage in more PA.
PO 4: Individuals make a plan specifying when, where and how to eat more fruit, more vegetables, or fewer high-energy snacks, or to engage in more PA.
PO 5: Individuals eat more fruit, more vegetables, or fewer high-energy snacks, or engage in more PA.
PO 6: Individuals evaluate if they managed to eat more fruit, more vegetables, or fewer high-energy snacks, or engage in more PA.
PO 7: If the goals have not been reached, individuals adjust their goal.

*PO* performance objective, *PA* physical activity

Determinants that may lead to the achievement of the performance objectives were derived from the literature. Determinants for behavioral change have been widely studied and suggested by theories such as self-regulation theories and the theory of planned behavior (TPB). Factors associated with diet and PA which are frequently described in the scientific literature include *awareness of one’s own risk behavior*, *knowledge*, *attitude*, *self-efficacy*, *social influences*, *intention*, *reevaluation of own behavior*, *goal setting* and *action planning* [45–51]. Focus group interviews among the target group showed that these determinants are important factors that may hinder or facilitate the behaviors in question. Furthermore, specific practical barriers and beliefs related to these determinants arose from the interviews, which will be addressed in our interventions. For instance, taste and health benefits were mentioned as attitude beliefs regarding healthy eating and PA. Barriers to eating healthily or engaging in PA included a lack of time and being too tired. For the Turkish and Moroccan participants, hospitality and religious factors were particularly mentioned as barriers to eating healthily or exercising (e.g. not being able to refuse food on social occasions, and men and women cannot exercise in the same location) [30]. In line with the IM procedure, the performance objectives and determinants were combined in a matrix. *Change objectives* for each performance objective-determinant combination were formulated to clarify what participants should learn as a result of the intervention, e.g.: *after having received the intervention, individuals state that eating more fruit is good*.

### Configuring intervention content and material

After having defined the behaviors and determinants to address, the intervention content was designed and two versions of a computer tailoring intervention were developed: one that includes tailoring to sociocognitive variables (traditional tailoring intervention) and one that additionally includes tailoring to the EI to contextualize the intervention material (EI-tailoring intervention).

**Fig. 1:**
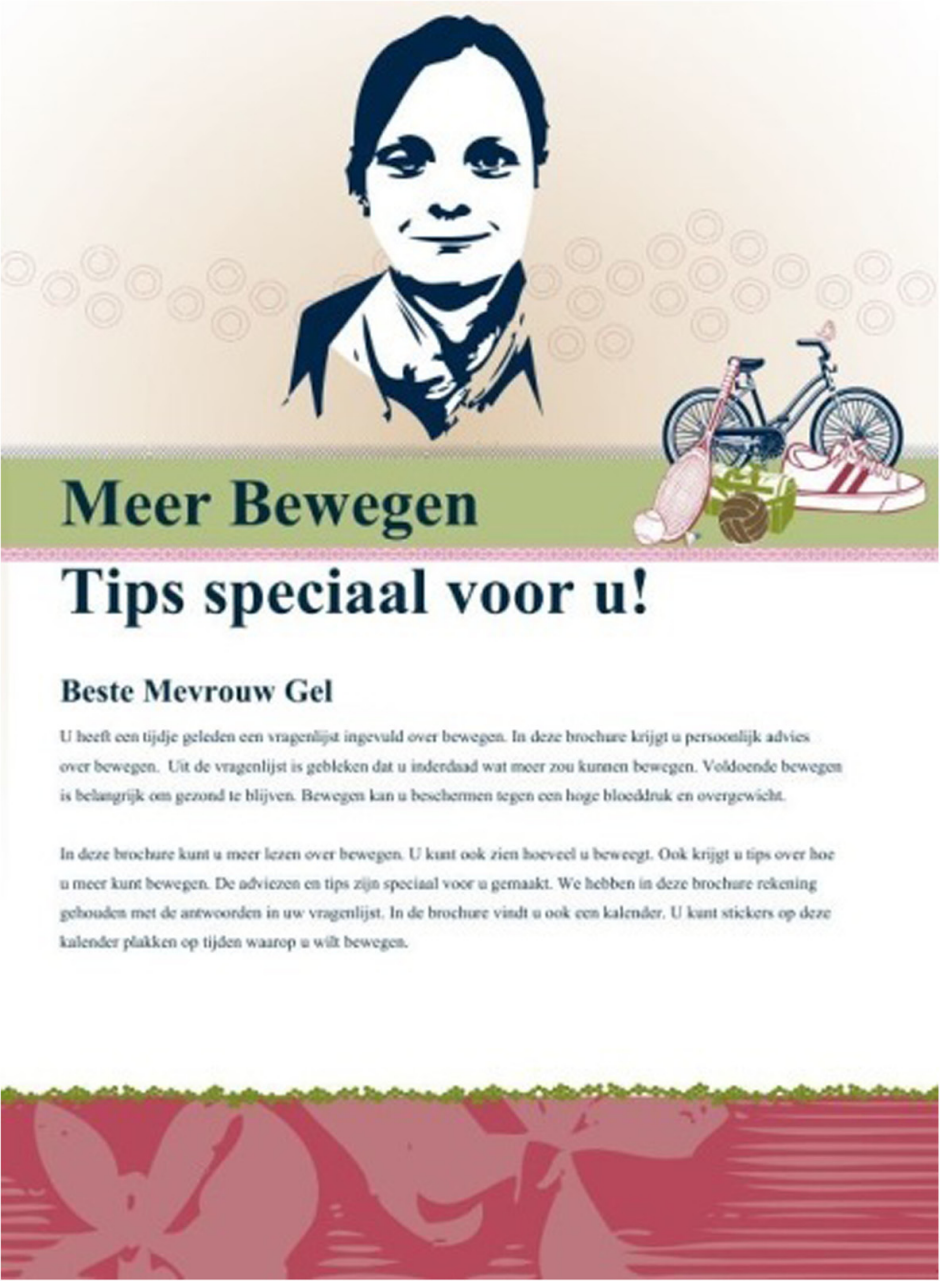
Title page of the traditional tailoring intervention, same for all ethnic groups

The traditional tailoring intervention consists of written feedback messages tailored to the recipients’ behavior and the above described determinants of the behavior. The feedback messages are delivered in a sequence of three printed brochures (see Fig. 1 for an example of a cover page of the traditional tailoring intervention). The first two brochures contain advice about two of the following behaviors: 1) fruit consumption, 2) vegetable consumption, 3) snack consumption, 4) PA. The recipient receives feedback on only two of the four behaviors, as addressing too many behaviors at a time may lead to ego depletion and because self-regulation might be a limited source [52, 53]. The third brochure is a follow-up brochure containing follow-up feedback on the behaviors addressed in the first two brochures. The advice messages in our interventions are partly based on existing computer tailoring programs [54, 55] and partly formulated by the intervention developers (i.e. the authors of this paper). We felt that print-delivered materials would work best for our target group, as during the focus group interviews that we conducted, some of the participants stated that they did not use, or infrequently used the Internet, and that print-delivered materials would be easier to use. Furthermore, materials delivered to the recipients’ home may be more noticeable. Research has shown that printed material may be used and recognized better than Web-based or computer-delivered interventions [56, 57]. Internet- and print-delivered health-promoting materials have both been shown to be effective [58].

#### Contextualizing the program material – tailoring to ethnic identity (EI)

We chose to use the concept of EI as a way to contextualize the intervention. EI is the degree to which someone identifies with his or her ethnic group of origin [42]. There are various instruments for measuring EI, such as the Multigroup Ethnic Identity Measure (MEIM) [59] and the revised version of the MEIM (MEIM-R) as described by Phinney and Ong [60]. An example item from the MEIM-R is: *I have a strong sense of belonging to my own ethnic group*.

For the target population of Turkish or Moroccan origin, we developed an EI scale based on the MEIM-R. We translated the items of the MEIM-R into Dutch and adjusted the phrasing to make them easier to understand for our lower-educated target group. Furthermore, we added the term “Turkish” or “Moroccan” to the items to make it clearer which ethnic group we are referring to. For instance, item 6) says: *I feel attached to Turkish people*. The original phrasing of item 6) is: *I feel a strong attachment towards my own ethnic group* [60]. The other items we used were: 1) *I find it important to know a lot about my Turkish/Moroccan origin, such as its history, traditions and customs*, 2) *Above all I feel Turkish/Moroccan*, 3) *It means a lot to me to be Turkish/Moroccan*, 4) *I find it important to understand what my Turkish/Moroccan origin involves*, and 5) *I often talk to other Turks/Moroccans about my origin to learn more about it*. Choosing a high score on these items means that someone is still very attached to their own ethnic group, which leads to allocation to category (1), i.e. having a strong Turkish/Moroccan EI. Medium scores lead to allocation to category (2), defined as having a medium Turkish/Moroccan EI, and low scores lead to being allocated to category (3), which is defined as having a weak Turkish/Moroccan EI and being more adapted to the Dutch ethnic group.

Contextualizing the content of intervention material by using EI tailoring can, for instance, be achieved by referring to the ethnic group in question in written advice messages, as shown by Resnicow et al. [42]. Contextualizing the design can be realized by using pictures of role models that match the EI of the individual, for instance pictures of a person from the same ethnic group [42]. A further way of contextualizing intervention material is by adjusting the layout of intervention material (e.g. colors, pictures and fonts) with the aim of developing culturally appropriate, interesting and credible intervention material for a target group [61]. We use EI-tailoring and culturally appropriate materials to make the intervention more attractive, noticeable and effective for the target group [41, 42, 61].

**Fig. 2:**
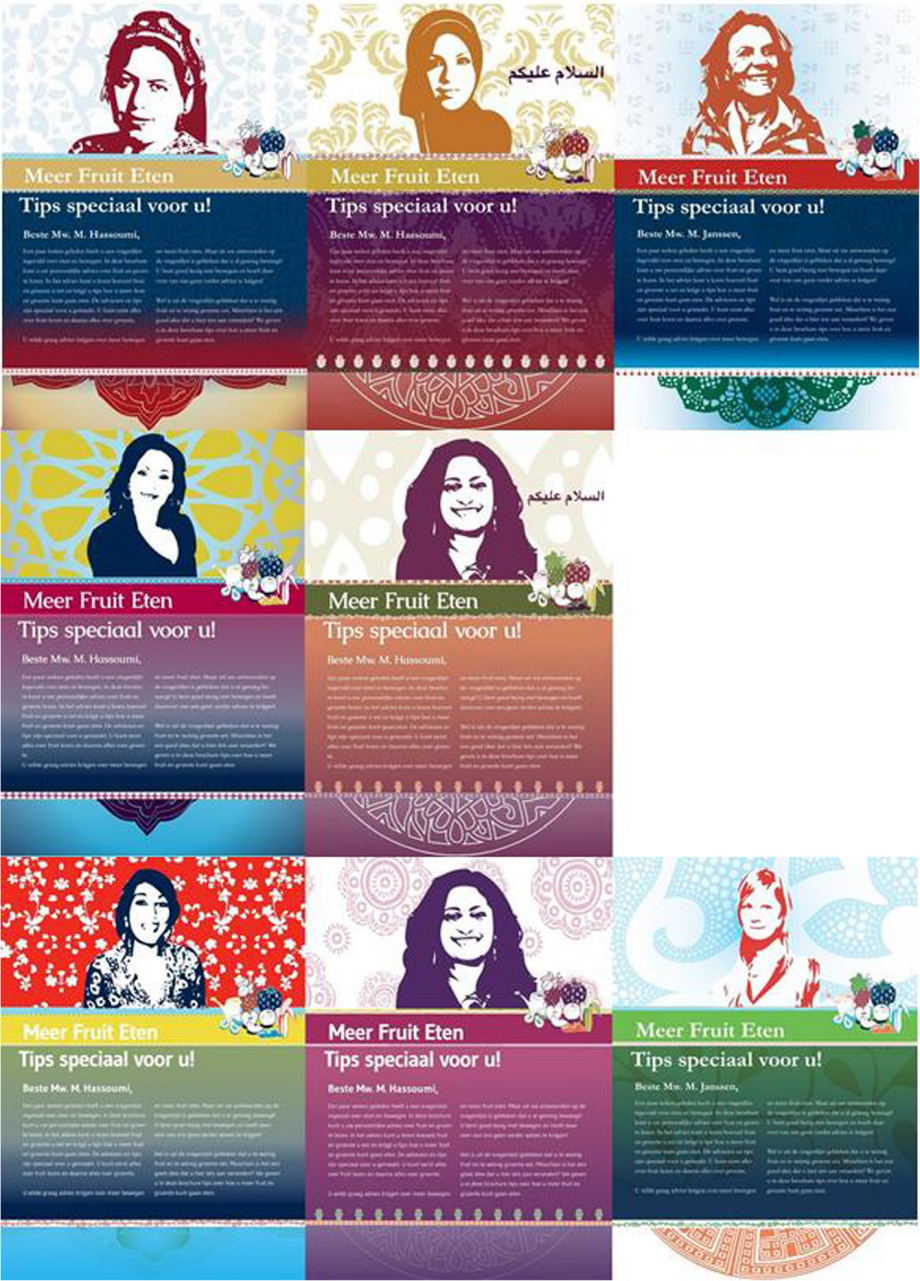
Title pages of the EI-tailoring intervention. The pictures show materials for Turkish, Moroccan and Dutch women (left to right), and for women with strong, medium and weak EI (top to bottom)

The messages and design of the intervention differ per EI category. For category (1), advice is provided that refers to the Turkish or Moroccan identity to a greater extent than the messages for categories (2) and (3). Messages for category (1) refer to the Turkish/Moroccan ethnic group, as well as their culture, such as food items and recipes, religious rules, cultural customs and traditions. In addition, for the brochures in category (1), traditional Turkish/Moroccan colors, patterns and symbols are used, as well as Turkish/Moroccan role models who have a traditional appearance. For category (2), the traditional cultural and religious rules, colors, patterns and symbols are applied to a lesser extent than for category (1). In category (3), the reference to the traditional culture was only applied minimally. The content of the brochures from this category are similar to the content of the traditional tailoring intervention. Moreover, a mix of Dutch and Turkish/Moroccan role models is used in categories (2) and (3). Figure 2 shows an example of how the lay-out, patterns and colors differ per EI category.

For the women of Dutch origin, we used a different EI-tailoring approach, as they live in their country of origin and are not exposed to a different ethnic group and culture they may adapt to. As the variation in EI within the Dutch group may be small, we differentiated between (1) “traditional” and (2) “modern” women of Dutch origin rather than having a strong, medium or weak EI. We chose to add this option to have a contextualized version for the women of Dutch origin as well.

Allocation to one of the categories is based on three items. Two of these items are inspired by research measuring the attitude concerning the family/sex role [62, 63] and the attitude towards women’s emancipation [64]. The third item is based on measurements of one’s attitude towards the in-group culture, such as traditional celebrations, music, language and food [42, 65]. An example item for measuring family-role attitudes is: *Husband and wife should share domestic tasks equally* [63]. Sex-role attitudes are measured in a similar way. An example item for the attitude towards women’s emancipation is: *A woman is more competent at raising small children than a man is* [64].

The items we formulated for our assessment are: 1) *I think that in my family mainly the woman should do the housework*, 2) *I think that in my family mainly the woman should take care of the parenting*, and 3) *I mainly eat Dutch dishes*. Higher scores on the items result in advice messages referring to Dutch traditions in eating and exercising, as well as how other Dutch women deal with certain situations. Messages for low-scoring people do not differ much from the standard messages in the traditional tailoring intervention, but they refer to a more modern way of life, food and exercising. Figure 2 shows how the layout of the brochures differs per EI category.

#### Intervention content and its underlying theoretical methods

The theory-driven methods that determined the content for our interventions derived from behavioral change theories, such as learning theories, goal-related theories and self-regulation theories [44]. The specific methods used in the two versions of the intervention are: *descriptive and evaluative feedback*, *active learning*, *provision of arguments*, *barrier identification* and *advice on how to deal with barriers*, *modeling*, *resistance to social pressure*, *stimulating communication to mobilize social support*, *goal setting*, *action planning* and *iterative feedback*. These methods will be outlined for each determinant in the following paragraph. We first describe the methods included in the first two brochures, followed by the methods applied in the third brochure. Tables 2 and 3 show example messages from the traditional tailoring intervention and the EI-tailoring intervention for the Turkish/Moroccan and the Dutch target groups, respectively.

**Table 2:** Example messages for the Turkish/Moroccan target group (comparing the traditional tailoring intervention and the EI-tailoring intervention, and the EI categories)

	Traditional tailoring intervention	EI-tailoring intervention		
		Strong T/M EI	Medium T/M EI	Weak T/M EI
Arguments for eating more fruit (why it is good to eat more fruit)	You do not think that eating fruit is healthy. But research shows that eating fruit is very good for your health […].	You do not think that eating fruit is healthy. But research shows that eating fruit is very good for your health […]. Looking after your health is also important in the Islamic faith.	You do not think that eating fruit is healthy. But research shows that eating fruit is very good for your health […]. Health is important in the Turkish/Moroccan and Dutch culture.	You do not think that eating fruit is healthy. But research shows that eating fruit is very good for your health […]. Health is important in the Dutch culture, but also in the Turkish/Moroccan culture.
How to deal with difficult situations	You find it difficult to eat fewer snacks when someone offers you a snack. […] Maybe you find it difficult or impolite to say ‘no’. But it is not impolite to say ‘no’ if you say that you want to consider your health and that you want to eat fewer snacks.	You find it difficult to eat fewer snacks when someone offers you a snack. […] In Turkey/Morocco it is common to offer food to visitors. It is then impolite to refuse. But maybe people will understand when you say that you want to consider your health and that you want to eat fewer snacks. […]	You find it difficult to eat fewer snacks when someone offers you a snack. […] In the Turkish/Moroccan and Dutch culture it is common to offer food to visitors. Maybe you find it impolite to refuse. But maybe people will understand when you say that you want to consider your health and that you want to eat fewer snacks. […]	You find it difficult to eat fewer snacks when someone offers you a snack. […] In the Turkish/Moroccan and Dutch culture it is common to offer food to visitors. Maybe you find it impolite to refuse. But maybe people will understand when you say that you want to consider your health and that you want to eat fewer snacks. […]

*T* Turkish, *M* Moroccan, *EI* ethnic identity

**Table 3:** Example messages for the Dutch target group (comparing the traditional tailoring intervention and the EI-tailoring intervention, and the EI categories)

	Traditional tailoring intervention	EI-tailoring intervention	
		Traditional	Modern
Arguments for eating more fruit (why fruit is an easy snack)	[…] There are many ways to make fruit a snack. Fruit is also a responsible snack. […]	[…] There are many ways to make fruit a snack. In the Dutch culture it is common to offer snacks with coffee. Fruit is then an easy choice. […]	[…] There are many ways to make fruit a snack. Fruit is, for instance, easy to take to work […].
How to deal with difficult situations	You find it difficult to eat fewer snacks when you are sad. […] But do you really feel better when you have eaten a lot? Try to find another solution. Maybe it helps to talk to someone. […]	You find it difficult to eat fewer snacks when you are sad. […] But do you really feel better when you have eaten a lot? Try to find another solution. Maybe it helps to talk to someone or be with your family. […]	You find it difficult to eat fewer snacks when you are sad. […] But do you really feel better when you have eaten a lot? Try to find another solution. Maybe it helps to talk to someone or be with your friends. […]

*EI* ethnic identity

#### Brochures one and two

The first two brochures each address one behavior (fruit consumption, vegetable consumption, snack consumption or PA) and its related determinants. The determinants addressed are *awareness of one’s own risk behavior*, *knowledge*, *attitude*, *self-efficacy*, *social influences*, *goal setting* and *action planning*.

An assessment tool is used to identify risk behaviors (i.e. not meeting the recommendations for the behaviors), accordingly, which two behaviors should be addressed in the brochures. The recipients can also choose two behaviors they would like to address. If they score unfavorably on more than two behaviors, their choice is taken into account. Moreover, the feedback addressing the behavioral determinants is tailored on the responses people have provided in the assessment tool.

#### Awareness

*Awareness of one’s own risk behavior* is increased by the method *descriptive and evaluative feedback* [41]. Each participant is provided with a feedback message indicating the quantity of fruit, vegetables or snacks she consumes, or how many minutes she is physically active for (descriptive feedback) and whether this is in line with the recommended level for the goal behavior (evaluative feedback). This information is provided in a brief text supported by a graphic showing whether the recipient sufficiently, partly or insufficiently meets the Dutch norm for the goal behavior. The same method is used in the EI-tailoring intervention, and no EI-tailoring is applied due to the complexity of the tailoring algorithms for this method.

#### Knowledge

The determinant *knowledge* is addressed with the method *active learning* [44]. This method is applied in the format of a list showing nine facts about diet or PA. Participants are asked to read these facts and check a box indicating whether or not they already know the fact. In the EI-tailoring intervention, facts are adjusted to the individual’s EI by referring to the ethnic group of origin (i.e. Dutch/Turkish/Moroccan women; women from the Netherlands/Turkey/Morocco) and to traditional food items, for instance by stating the amount of calories a piece of baklava contains.

#### Attitude

In the tailored feedback that addresses the *attitude* towards the goal behavior, *arguments* [44] are provided in the form of text messages. These messages aim to reinforce advantages and to refute disadvantages the recipient perceives regarding the goal behavior. Additionally, an illustration of a physician as a credible source is presented to emphasize the message content. In the EI-tailoring intervention, the messages refer to the ethnic group, food items and cultural rules and customs. For instance, some of the messages refer to the importance in Islam of taking care of one’s health. Furthermore, a Turkish or Moroccan physician is shown in the picture. For the traditional Dutch women, the messages refer to Dutch customs and traditions, such as the fact that cycling is a common habit in the Dutch population and how fruit and vegetables are important in the Dutch food culture.

#### Self-efficacy and social influence

The methods used to improve the determinants *self-efficacy* and *social influence* are combined by integrating the advice for *social influences* into the advice messages that address *self-efficacy. Self-efficacy* is addressed with several methods. First, the target group is stimulated to *identify barriers* that prevent them from carrying out the healthy behavior, by indicating difficult situations in the assessment tool [66]. Then *advice is provided* on how to deal with these barriers. A further method is *modeling*, which is realized by including role model stories in the advice to show how others deal with difficult situations. The text is accompanied by a picture of a role model that is tailored to the individual’s age.

The determinant *social influence* is addressed by providing tips on how to *resist social pressure* to eat unhealthy food or to be physically inactive [44]. Moreover, advice is given on how to *communicate to mobilize social support* [44]. The individual can, for instance, ask family and friends to help them perform the goal behavior.

The messages and pictures are additionally matched with the individual’s EI in the EI-tailoring intervention. For the more traditional women, the messages refer, for instance, to traditional dishes and to the fact that Turkish and Moroccan dishes contain a lot of vegetables. In addition, the role model pictures and names refer to women with a traditional appearance compared to the more modern appearance of the women in the brochures for the less traditional women.

#### Goal setting and action planning

*Goal setting* and *action planning* are important determinants and at the same time methods to stimulate behavior change and to bridge the intention-behavior gap [45, 66]. *Goal setting* is applied by providing information about the importance of setting a specific goal to reach the goal behavior. Moreover, a list with goals the individual can choose from is included. The goals are provided in a closed-ended format and the recipient can mark one goal they want to achieve. We decided to let the recipient choose a goal instead of writing down an own goal to simplify the intervention for the lower-educated target group and make the goal-setting easier.

**Fig. 3:**
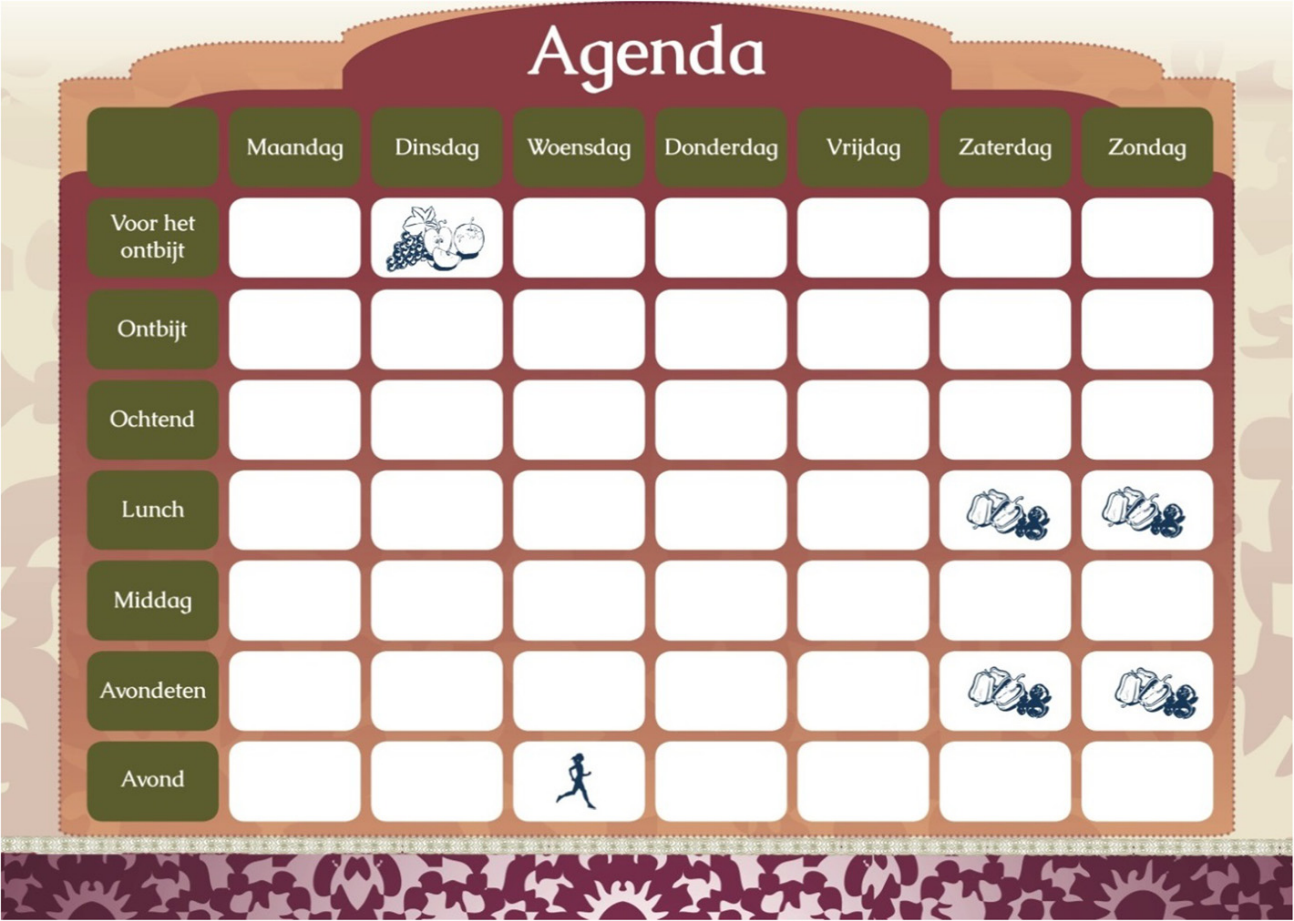
Planning agenda. The headings at the top are the days of the week (Monday to Sunday); the boxes from top to bottom represent seven time periods of the day: before breakfast, breakfast, morning, lunch, afternoon, dinner, evening

To stimulate *action planning*, a page is added to the brochure containing a week’s agenda that the individual can use to plan the specific actions they want to take to achieve their goal (see Fig. 3). The agenda is accompanied with stickers that can be used to indicate on which days and at what time the individual wants to take a specific action (e.g. perform a physical activity, eat a piece of fruit). Furthermore, a list with four preparatory actions is provided in the brochure that stimulates the individual to use the agenda (e.g. stick the stickers on your agenda on the days you want to exercise). *Goal setting* and *action planning* are applied in the EI-tailoring intervention in the same way (no EI-tailoring is applied). The layout of the agenda, however, differs for each EI category in terms of colors.

#### Brochure three

The follow-up brochure contains advice about both behaviors addressed in the previous brochures and addresses the determinants of the last phase of self-regulation (*volitional phase*). These determinants are: *evaluation of progress* of own behavior change, *self-efficacy*, *social influence*, *goal setting* and *action planning*. Table 4 shows a full overview of the methods used in the brochures. A second measurement is conducted to receive input for the follow-up brochure. The same assessment tool as for the first two brochures is used to measure behavior and its determinants.

**Table 4:** Theory-based methods and practical applications per determinant (motivational and volitional phase)

Determinant	Method^a^	Theoretical/empirical foundation	Parameter for use	Practical application
Motivational phase				
Awareness	Descriptive and evaluative feedback	[41]	Feedback to raise awareness should be followed by increase in problem solving ability and self-efficacy	Written message reflecting the recipient’s health behavior Graphic indicating whether the recipient adheres to the norm of the health behavior (traffic light system)
Knowledge	Active learning	[82]	Time, information and skills	Written facts about fruit, vegetables, snacks or PA Interactive element included
Attitude	Arguments	[83–85]	The arguments should be new to the individuals	Written messages providing arguments for the goal behavior An image of a credible role model is used
Self-efficacy	Barrier identification and advice on how to deal with barriers	[66]	Target group should identify high-risk situations: List with difficult situations (barriers) in the assessment tool from which the target group can choose	Written advice on how to deal with difficult situations
	Modeling	[86]	Attention, remembrance, skills, reinforcement; the source, method and channel should be credible	Role model stories and an image of a role model
Social influence	Resistance to social pressure	[87]	Commitment to intention is required, psychological inoculation against pressure	Written messages to stimulate the recipient to resist social pressure
	Stimulate communication to mobilize social support	[88]	Support should be available in the environment	Written messages to stimulate the recipient to ask for support in their environment
Volitional phase				
Evaluation of progress	Iterative feedback	[67]	-	Written message reflecting whether behavior change was achieved Graphics indicating whether the recipient adheres to the norm of the health behavior (traffic light system), and whether there was a change
Goal setting	Goal setting	[45]	Target group should be committed to the goal, and goals should be difficult but feasible	List of goals from which the recipient can choose a personal goal
Action planning	Planning	[66]	Target group should have decided to make a change and have the intention to change	List of planning strategies the recipient can follow Planning tool: Recipients can plan for every day of the week when they want to perform the goal behavior

^a^Most methods and parameters for use were adopted from Bartholomew et al. [44]

#### Evaluation of progress

The follow-up brochure aims to stimulate the recipient to evaluate the progress in their own behavior after having received the first two brochures. This is achieved by comparing the behavior at the follow-up assessment with the behavior at the baseline assessment, which is based on the *iterative feedback* principle [67]. A short text message is provided describing to the recipient whether or not she achieved a change. The text is accompanied by two graphics showing whether the individual sufficiently, partly or insufficiently meets the Dutch norm for the goal behavior at the baseline assessment and at the follow-up assessment. The same method is used in the EI-tailoring intervention, and no EI-tailoring is applied due to the complexity of the tailoring algorithms for this method.

#### Self-efficacy and social influence

To further improve the recipients’ *self-efficacy* in the volitional phase, they again choose two difficult situations. Also *social influence* is addressed again. The same methods are used as in the first brochures. The content of the advice differs from the advice in the first two brochures to give the recipients new options on how to deal with difficult situations and to prevent repetition of the same advice from the first two brochures.

#### Goal setting and action planning

The same methods as for the first two brochures are used to bridge the intention-behavior gap. The recipient can choose to go on with the same goal or to adjust the goal by choosing a new one. *Planning* is applied by asking the individual to plan the goal with the same agenda from the previous brochures and by using the same preparatory actions as described above.

#### Scope and sequence of the brochures

**Fig. 4:**
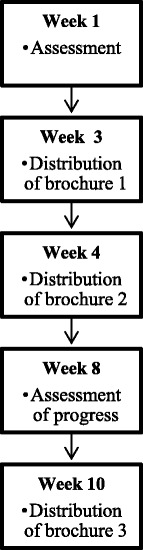
Sequence of the intervention delivery

Two measurement moments take place during the intervention, one before the intervention and one after the recipient has received the first two brochures (see Fig. 4). The first two brochures start with a cover page containing a short introduction, and a colophon. From page three onward, the determinants are addressed in an order that is in line with the self-regulation process. The first few pages focus on the *motivational phase*: Page three addresses the determinant *awareness*, page four focuses on the determinant *knowledge*, page five is aimed at the determinant *attitude* and page six at the determinants *self-efficacy* and *social influence*. Pages seven and eight address *goal setting* and *action planning*. The follow-up brochure starts with a cover page introducing the brochure. Then, the recipients receive feedback on their own progress in the first behavior, followed by tips to improve self-efficacy and social influences. Lastly, goal setting and action planning are stimulated. The same is repeated for the second behavior. The last page contains a healthy recipe or a description of a simple exercise that can be carried out at home.

#### The technology behind computer tailoring

To tailor the health messages to the specific beliefs and behavior of the target group, the computer tailoring program TailorBuilder was used (featured by OverNite Software Europe). Logical algorithms are used in this online tool to produce personalized feedback for the recipients of the interventions. By means of these algorithms, the individual receives advice messages from a set of previously formulated messages that match with the questionnaire scores on behavior and sociocognitive beliefs regarding the behavior.

**Fig. 5:**
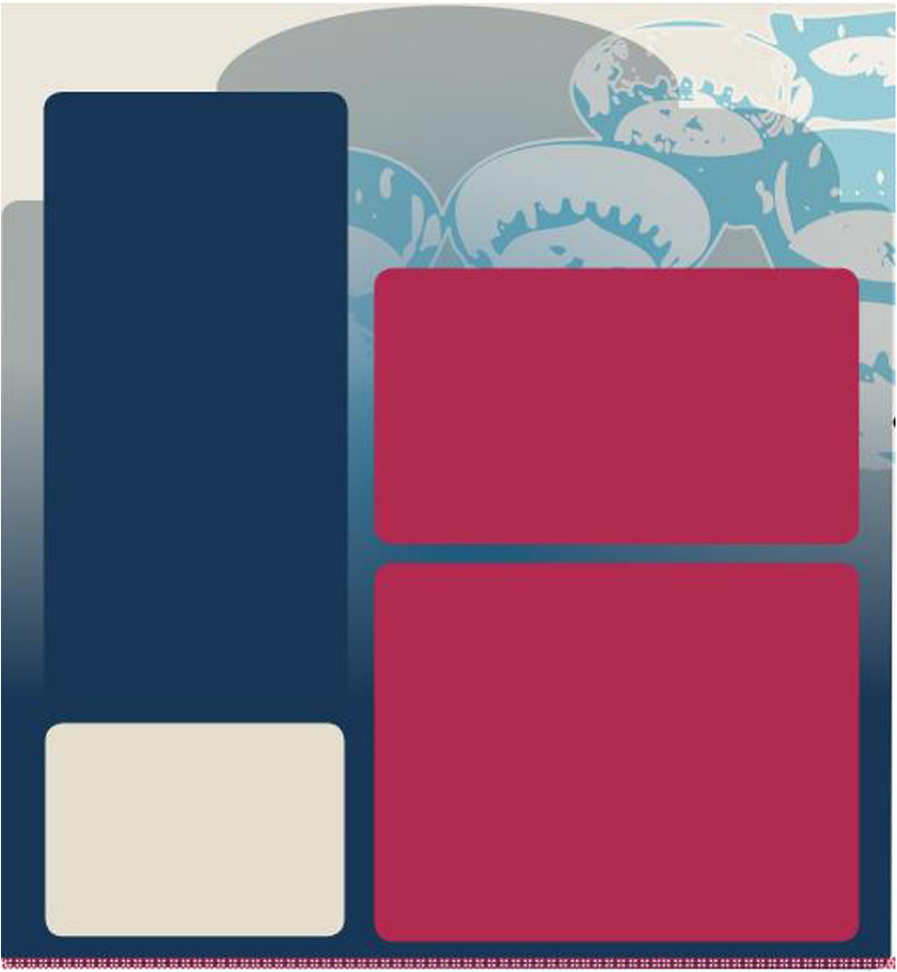
Example page from brochure template

The layout of the intervention material was designed by a graphic designer. Several templates were designed for each EI category and one for the traditional tailoring intervention and the control intervention. The templates were delivered without the text messages. These were inserted later in the process using TailorBuilder. Figure 5 shows an example page from the templates.

#### Pretest of the intervention material

Both versions of the intervention were pretested in order to be able to adapt the content. There were two phases during the pretest. In the first phase, tentative drafts of the intervention materials were shown to women from the target group. The women were asked to evaluate draft advice messages, pictures used in the brochures and the layout of the brochures. A qualitative approach was applied by asking the participants to read through the material, while an interviewer asked questions about the likability, readability, attractiveness and usability of the material. During the second phase of the pretest, the intervention delivery process was assessed. Five Dutch women (mean age 55.8 years) and 18 Turkish women (mean age 41.8 years) filled out the baseline questionnaire and received the first two brochures, which were tailored to their answers on the questionnaire. The participants were asked to give feedback on the brochures as a whole by means of a structured questionnaire asking, for instance, about the degree of personal relevance and individualization of the brochures.

Generally, the intervention material was appreciated by the women. They perceived the texts as readable and interesting and both the length of the messages and the layout were appropriate. The material was perceived as personally relevant and the women said that they would use the advice given in the brochures. General critique points were that the pictures of the role models and vegetables could be improved by making them more precise. A few women said that the information in the brochure was not new to them and one woman would not use the agenda that is provided with the brochure because it would take her too much time. The Turkish women criticized strongly the way the brochures for the EI-tailoring intervention referred to religious rules, which they thought was offensive and irrelevant. Furthermore, they did not like the fact that all of the role models in the picture were wearing a headscarf in the traditional EI category. Therefore, we decided to remove parts of the text and images that referred to religious rules and Islam from the EI-tailoring intervention for the Turkish population.

### Evaluating the interventions

In the fifth and sixth steps of the IM protocol, the implementation and evaluation of the intervention are planned [44]. Before starting implementation efforts, we will evaluate the intervention’s effectiveness.

#### Design, participants and procedure

The two versions of the intervention will be evaluated in a randomized controlled trial with three study groups: traditional tailoring, EI-tailoring and a control group receiving generic, nontailored information. The primary outcome measures are fruit, vegetable and snack consumption, and PA. Mediating variables are awareness of one’s own behavior, attitude, self-efficacy, social support and intention towards more fruit, vegetable and snack consumption and PA. A process evaluation will be conducted at three-month follow-up, assessing the self-reported use, relevance and appreciation of the intervention.

It is hypothesized that both interventions are more effective than the control intervention and that the EI-tailoring intervention will reach higher effect sizes and higher acceptance among the target group than the traditional tailoring intervention. The required number of participants for this study is 1000, of which 500 should be of Dutch origin, 250 of Turkish origin and 250 of Moroccan origin. To be able to detect a small intervention effect (ES = 0.3), with a power of 0.8 and a significance level of p < .05, 250 people are needed in each study condition, which adds up to 750 participants in total. Taking a dropout rate of 10 % at each measurement moment into account, 1000 participants have to be included at baseline.

The target group consists of lower-educated adult women (lower vocational level or below), who are aged between 20 and 65 years, and who are of Dutch, Turkish or Moroccan origin. We use the standard definition for origin as described by Statistics Netherlands [68]. Participants with an insufficient knowledge of the Dutch language, or a health condition that may restrict their eating and/or PA behavior, are excluded from the study. Moreover, women whose baseline assessment shows that they do not perform the risk behaviors will be excluded from the study.

We use active recruitment strategies by approaching participants directly. In addition, passive strategies are applied by sending, for instance, e-mails to potential participants. Table 5 provides an overview of all recruitment strategies. The paper questionnaires will be sent to the participants’ home address or handed out to them in person. After the baseline measurement, participants will be pre-stratified based on their origin. The participants will then be randomly assigned to one of the three intervention groups by means of a computer-determined sequence. The intervention material is generated by means of the TailorBuilder program. The brochures will be sent or handed out to the participants. The participants will be rewarded with gift vouchers worth €20.

**Table 5:** Recruitment strategies

Active recruitment strategies
Approaching participants via general practitioners or nurse practitioners
Approaching participants at events, markets and fairs
Approaching participants at public venues, such as community centers and mosques
Approaching participants via foundations or social work organizations
Approaching participants via stakeholders from the target group
Approaching participants via the public health service
Word-to-mouth distribution via participants (snow ball method)
Approaching participants via research/recruitment institutes (active approach by Turkish and Moroccan recruiters)
Passive recruitment strategies
Call for participation via public media -Websites -Magazines -Facebook
Call for participation via companies via e-mail -Cleaning companies -Nursing companies -Home care companies
Call for participation via research panels from research/recruitment institutes via e-mail

#### Measurements

**Fig. 6:**
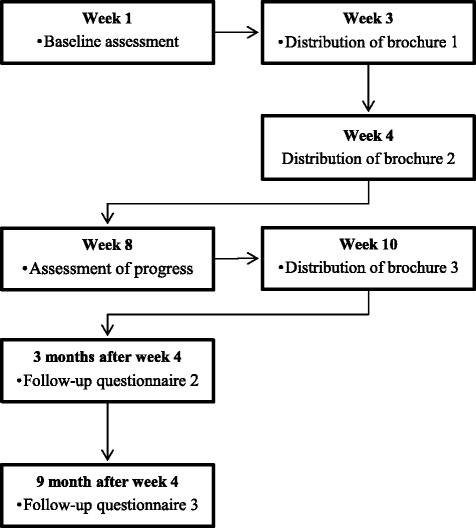
Flow-chart of measurement moments and intervention procedure

Measurement moments will be at baseline, one month, three months and nine months post-intervention, which is after participants have received the first two brochures of the intervention (see Fig. 6 for a flow-chart). A food frequency questionnaire based on validated measurement tools is used to measure the fruit, vegetable and snack intake [69–71]. An adapted version of the Short Questionnaire to Assess Health-enhancing Physical Activity (SQUASH) will be used to measure PA [72]. Furthermore, items measuring the sociocognitive determinants of dietary and PA behavior will be used (i.e. awareness of one’s own behavior, attitude, self-efficacy, social support and intention) which are based on previous studies and are adjusted to our target population [73–76]. The process will be evaluated by means of process variables used in previous research [77].

#### Statistical analysis

Multivariable linear and logistic regression analysis will be conducted to assess group differences in the fruit, vegetable and snack intake and PA at different points in time. Moderation analysis will be conducted to assess whether the intervention effects are moderated by demographic factors, such as ethnic background or EI. Further, mediation analysis will be conducted to identify the factors that may explain the intervention effect (e.g. attitude and self-efficacy).

#### Ethical approval

This study was submitted to the committee for ethical evaluation of research at the Open University in Heerlen, the Netherlands (commissie Ethische Toetsing Onderzoek, cETO). According to the cETO, this RCT does not come under the WMO law (Wet maatschappelijke ondersteuning/Social support act). Therefore, ethical approval of the Regional Medical Ethics committee in the Netherlands was not necessary for this project. The RCT will, however, be conducted according to good research practice by informing participants about the study and by asking for their informed consent to participate. This study is registered with the Nederlands Trial Register (NTR: 4506).

## Discussion

The aim of this paper was to provide a detailed description of the development of two versions of a computer-tailored intervention aimed at improving dietary and PA behavior among Dutch, Turkish and Moroccan adult women living in the Netherlands. In the first version of the intervention, content matching is applied by tailoring to behavior and sociocognitive factors (e.g. awareness, attitude and self-efficacy). In the second version, the materials are additionally contextualized by tailoring the material to EI.

We used the intervention mapping (IM) protocol to develop a theory- and evidence-based intervention. IM has often been used to develop nutrition- and/or PA-improving interventions before, and was evaluated as a good and useful tool for systematically developing such interventions [78–80]. However, we encountered challenges during the intervention development process related to choosing determinants and methods for behavioral change among this specific target group. As little evidence is available regarding determinants of dietary and PA behavior for lower-educated and various ethnic groups, we cannot be sure that we chose the most important determinants. The same applies for the techniques we used in the intervention to initiate behavior change. These methods have not been tested among Turkish and Moroccan women in the Netherlands before. Preliminary research among our target group led us to make use of the same determinants and behavior change techniques, as the differences between the ethnic groups were minimal [30].

A further challenge in this project is to reach the lower-educated and ethnic-minority populations, since they have emerged as difficult target groups to recruit for scientific research [81]. Current interventions aimed at lower-educated and ethnic-minority populations are often implemented on a small scale. By making use of computer tailoring we developed an intervention that can reach large numbers of people, and still provide individual advice. This paper illustrates how intervention material can be contextualized using EI-tailoring and cultural appropriateness. EI-tailoring has been proven to be effective among African American populations in the U.S. [42]. A randomized controlled trial will show whether the interventions are effective among our specific target group and whether EI-tailoring is beneficial.

## References

[1] Sjöström M, Oja P, Hagströmer M, Smith BJ, Bauman A (2006). Health-enhancing physical activity across European Union countries: the Eurobarometer study. J Public Health.

[2] Subar AF, Heimendinger J, Patterson BH, Krebs-Smith SM, Pivonka E, Kessler R (1995). Fruit and vegetable intake in the United States: the baseline survey of the Five A Day for Better Health Program. Am J Health Promot.

[3] Naska A, Vasdekis VGS, Trichopoulou A, Friel S, Leonhäuser IU, Moreiras O, Nelson M, Remaut AM, Schmitt A, Sekula W (2000). Fruit and vegetable availability among ten European countries:how does it compare with the ‘five-a-day’ recommendation?. Br J Nutr.

[4] Ball K, Crawford D, Mishra G (2006). Socio-economic inequalities in women’s fruit and vegetable intakes: a multilevel study of individual, social and environmental mediators. Public Health Nutr.

[5] Hulshof KFAM, Brussaard JH, Kruizinga AG, Telman J, Löwik MRH (2003). Socio-economic status, dietary intake and 10 y trends: the Dutch National Food Consumption Survey. Eur J Clin Nutr.

[6] De Irala-Estévez J, Groth M, Johansson L, Prättälä R, Martínez- González MA (2000). A systematic review of socio-economic differences in food habits in Europe: consumption of fruit and vegetables. Eur J Clin Nutr.

[7] Hildebrandt VH, Bernaards CM, Stubbe JH (2013). Trendrapport Bewegen en Gezondheid 2010/2011.

[8] Breedveld K, Kamphuis C, Tiessen-Raaphorst A (2008). Rapportage Sport 2008.

[9] August K, Sorkin D (2011). Racial/ethnic disparities in exercise and dietary behaviors of middle-aged and older adults. J Gen Intern Med.

[10] Roos G, Johansson L, Kasmel A, Klumbiené J, Prättälä R (2001). Disparities in vegetable and fruit consumption: European cases from the north to the south. Public Health Nutr.

[11] Zizza C, Siega-Riz AM, Popkin BM (2001). Significant increase in young Adults’ snacking between 1977–1978 and 1994–1996 represents a cause for concern!. Prev Med.

[12] Kant AK, Graubard BI (2006). Secular trends in patterns of self-reported food consumption of adult Americans: NHANES 1971-1975 to NHANES 1999–2002. Am J Clin Nutr.

[13] Tucker JM, Welk GJ, Beyler NK (2011). Physical activity in U.S. adults: compliance with the physical activity guidelines for americans. Am J Prev Med.

[14] Pomerleau J, McKee M, Lobstein T, Knai C (2003). The burden of disease attributable to nutrition in Europe. Public Health Nutr.

[15] Hu FB, Manson JE, Stampfer MJ, Colditz G, Liu S, Solomon CG, Willett WC (2001). Diet, lifestyle, and the risk of type 2 diabetes mellitus in women. N Engl J Med.

[16] Martinez-Gonzalez MA, Varo JJ, Santos JL, Irala J, Gibney M, Kearney J, Martinez JA (2001). Prevalence of physical activity during leisure time in the European Union. Med Sci Sports Exerc.

[17] Sorkin DH, Billimek J (2012). Dietary behaviors of a racially and ethnically diverse sample of overweight and obese Californians. Health Educ Behav.

[18] Lee SH, Im E-O (2010). Ethnic differences in exercise and leisure time physical activity among midlife women. J Adv Nurs.

[19] Van Rossum C, Fransen H, Verkaik-Kloosterman J, Buurma-Rethans E, Ocké M. Dutch national food consumption survey 2007-2010: diet of children and adults aged 7 to 69 years. RIVM rapport 350050006. 2011.

[20] Gezondheidsraad (2015). Richtlijnen goede voeding 2015.

[21] Brink L, Postma-Smeets A, Stafleu A, Wolvers D (2016). Richtlijnen schijf van vijf.

[22] Hupkens CL, Knibbe RA, Drop MJ (2000). Social class differences in food consumption. Eur J Public Health.

[23] CBS. Bevolking; generatie, geslacht, leeftijd en herkomstgroepering, 1 januari [http://statline.cbs.nl/StatWeb/publication/?DM=SLNL&PA=37325&D1=0&D2=0&D3=0&D4=0&D5=a&D6=l&HDR=T,G2,G3,G5&STB=G1,G4&VW=T]. 09-02-2015

[24] Cornelisse-Vermaat JR, van den Brink HM (2007). Ethnic differences in lifestyle and overweight in the Netherlands. Obesity (Silver Spring).

[25] Palsma A, Nicolau M, van Dam R, Stronks K (2006). De voeding van Turkse en Marokkaanse Nederlanders in de leeftijd van 18-30 jaar. Prioriteiten voor voedingsinterventies. Tijdschr Sociale Geneesk.

[26] Van ‘t Riet H, Dijkshorn H, Corstjens R, Berkouwer L (2005). Gezonde Leefgewoonten Westerpark; Kwalitatief interventieonderzoek naar overgewicht bij Turkse en Marokkaanse vrouwen van 25 tot 45 jaar. Probleemanalyse.

[27] Dowler E (2001). Inequalities in diet and physical activity in Europe. Public Health Nutr.

[28] Ball K, Timperio A, Salmon J, Giles-Corti B, Roberts R, Crawford D (2007). Personal, social and environmental determinants of educational inequalities in walking: a multilevel study. J Epidemiol Community Health.

[29] De Boer EJ, Brants HAM, Beukers M, Ocké MC, Dekker L, Nicolaou M, Snijder M (2015). Voeding van Marokkaanse, Turkse, Surinaamse en autochtone Nederlanders in Amsterdam. RIVM rapport 2015-0099.

[30] Romeike K, Abidi L, Lechner L, de Vries H, Oenema A (2016). Similarities and differences in underlying beliefs of socio-cognitive factors related to diet and physical activity in lower-educated Dutch, Turkish, and Moroccan adults in the Netherlands: a focus group study. BMC Public Health.

[31] van Erp-Baart MA, Westenbrink S, Hulshof KF, Brussaard JH (2001). Assessment of dietary intake among Moroccan women and Surinam men. Ethn Health.

[32] Noar SM, Benac CN, Harris MS (2007). Does tailoring matter? Meta-analytic review of tailored print health behavior change interventions. Psychol Bull.

[33] Park E-J, McDaniel A, Jung M-S (2009). Computerized tailoring of health information. Comput Inform Nurs.

[34] Kroeze W, Werkman A, Brug J (2006). A systematic review of randomized trials on the effectiveness of computer-tailored education on physical activity and dietary behaviors. Ann Behav Med.

[35] Smeets T, Kremers SPJ, Brug J, de Vries H (2007). Effects of tailored feedback on multiple health behaviors. Ann Behav Med.

[36] De Bourdeaudhuij I, Brug J (2000). Tailoring dietary feedback to reduce fat intake: an intervention at the family level. Health Educ Res.

[37] de Vries H, Kremers SP, Smeets T, Brug J, Eijmael K. The effectiveness of tailored feedback and action plans in an intervention addressing multiple health behaviors. Am J Health Promot. 2008;22. 10.4278/ajhp.22.6.417 18677882

[38] Walthouwer MJL, Oenema A, Lechner L, de Vries H (2015). Use and effectiveness of a video-and text-driven Web-based computer-tailored intervention: randomized controlled trial. J Med Internet Res.

[39] Springvloet L, Lechner L, de Vries H, Candel MJ, Oenema A (2015). Short-and Medium-Term Efficacy of a Web-Based Computer-Tailored Nutrition Education Intervention for Adults Including Cognitive and Environmental Feedback: Randomized Controlled Trial. J Med Internet Res.

[40] Peels DA, Bolman C, Golsteijn RHJ, de Vries H, Mudde AN, van Stralen MM, Lechner L (2013). Long-term efficacy of a printed or a Web-based tailored physical activity intervention among older adults. Int J Behav Nutr Phys Act.

[41] Hawkins RP, Kreuter M, Resnicow K, Fishbein M, Dijkstra A (2008). Understanding tailoring in communicating about health. Health Educ Res.

[42] Resnicow K, Davis R, Zhang N, Tolsma D, Alexander G, Wiese C, Cross WE, Anderson JP, Calvi J, Strecher V (2009). Tailoring a fruit and vegetable intervention on ethnic identity: results of a randomized study. Health Psychol.

[43] Kreuter MW, Sugg-Skinner C, Holt CL, Clark EM, Haire-Joshu D, Fu Q, Booker AC, Steger-May K, Bucholtz D (2005). Cultural tailoring for mammography and fruit and vegetable intake among low-income African-American women in urban public health centers. Prev Med.

[44] Bartholomew LK, Parcel GS, Kok G, Gottlieb NH, Fernández ME (2011). Planning health promotion programs.

[45] Maes S, Karoly P (2005). Self-regulation assessment and intervention in physical health and illness: a review. Appl Psychol.

[46] Blue CL, Wilbur J, Marston‐Scott MV (2001). Exercise among blue‐collar workers: application of the theory of planned behavior. Res Nurs Health.

[47] Blanchard CM, Fisher J, Sparling PB, Shanks TH, Nehl E, Rhodes RE, Courneya KS, Baker F (2009). Understanding adherence to 5 servings of fruits and vegetables per day: a theory of planned behavior perspective. J Nutr Educ Behav.

[48] Bogers R, Brug J, van Assema P, Dagnelie P (2004). Explaining fruit and vegetable consumption: the theory of planned behaviour and misconception of personal intake levels. Appetite.

[49] Ajzen I (1991). The theory of planned behavior. Organ Behav Hum Decis Process.

[50] Shaikh AR, Yaroch AL, Nebeling L, Yeh M-C, Resnicow K (2008). Psychosocial predictors of fruit and vegetable consumption in adults: a review of the literature. Am J Prev Med.

[51] Guillaumie L, Godin G, Vézina-Im L-A (2010). Psychosocial determinants of fruit and vegetable intake in adult population: a systematic review. IJBNPA.

[52] Baumeister RF, Bratslavsky E, Muraven M, Tice DM (1998). Ego depletion: is the active self a limited resource?. J Pers Soc Psychol.

[53] Hagger MS, Wood C, Stiff C, Chatzisarantis NL (2010). Ego depletion and the strength model of self-control: a meta-analysis. Psychol Bull.

[54] Springvloet L, Lechner L, Oenema A (2014). Planned development and evaluation protocol of two versions of a web-based computer-tailored nutrition education intervention aimed at adults, including cognitive and environmental feedback. BMC Public Health.

[55] Walthouwer MJ, Oenema A, Soetens K, Lechner L, De Vries H (2013). Systematic development of a text-driven and a video-driven web-based computer-tailored obesity prevention intervention. BMC Public Health.

[56] Kroeze W, Oenema A, Campbell M, Brug H. Comparison of use and appreciation of a print-delivered versus CD-ROM-delivered, computer-tailored intervention targeting saturated fat intake: Randomized controlled trial. J Med Internet Res 2008;10(2):e12. 10.2196/jmir.940 PMC2483920 18487136

[57] Marshall AL, Leslie ER, Bauman AE, Marcus BH, Owen N (2003). Print versus website physical activity programs: A randomized trial. Am J Prev Med.

[58] Kroeze W, Oenema A, Campbell M, Brug J (2008). The Efficacy of Web-based and Print-delivered Computer-tailored Interventions to Reduce Fat Intake: Results of a Randomized. Controlled Trial J Nutr Educ Behav.

[59] Ponterotto JG, Gretchen D, Utsey SO, Stracuzzi T, Saya R (2003). The multigroup ethnic identity measure (MEIM): Psychometric review and further validity testing. Educ Psychol Meas.

[60] Phinney JS, Ong AD (2007). Conceptualization and measurement of ethnic identity: Current status and future directions. J Couns Psychol.

[61] Kreuter MW, Lukwago SN, Bucholtz DC, Clark EM, Sanders-Thompson V (2003). Achieving cultural appropriateness in health promotion programs: targeted and tailored approaches. Health Educ Behav.

[62] Kalmijn M (2005). Attitude alignment in marriage and cohabitation: The case of sex‐role attitudes. Pers Relatsh.

[63] Kraaykamp G (2012). Employment status and family role attitudes: A trend analysis for the Netherlands. Int Sociol.

[64] Neve RJM (1995). Changes in attitudes toward Women’s emancipation in the Netherlands over Two decades: unraveling a trend. Soc Sci Res.

[65] Phinney JS (1990). Ethnic identity in adolescents and adults: review of research. Psychol Bull.

[66] Michie S, Ashford S, Sniehotta FF, Dombrowski SU, Bishop A, French DP (2011). A refined taxonomy of behaviour change techniques to help people change their physical activity and healthy eating behaviours: the CALO-RE taxonomy. Psychol Health.

[67] Brug J, Glanz K, Van Assema P, Kok G, Van Breukelen GJ (1998). The impact of computer-tailored feedback and iterative feedback on fat, fruit, and vegetable intake. Health Educ Behav.

[68] CBS (2000). Maandstatistiek van de bevolking.

[69] Van Assema P, Brug J, Ronda G, Steenhuis I, Oenema A (2002). A short Dutch questionnaire to measure fruit and vegetable intake: relative validity among adults and adolescents. Nutr Health.

[70] Van Assema P, Brug J, Ronda G, Steenhuis I (2001). The relative validity of a short Dutch questionnaire as a means to categorize adults and adolescents to total and saturated fat intake. J Hum Nutr Diet.

[71] Beukers MH, Dekker LH, de Boer EJ, Perenboom CWM, Meijboom S, Nicolaou M, de Vries JHM, Brants HAM (2015). Development of the HELIUS food frequency questionnaires: ethnic-specific questionnaires to assess the diet of a multiethnic population in The Netherlands. Eur J Clin Nutr.

[72] Wendel-Vos GW, Schuit AJ, Saris WH, Kromhout D (2003). Reproducibility and relative validity of the short questionnaire to assess health-enhancing physical activity. J Clin Epidemiol.

[73] Walthouwer MJL, Oenema A, Lechner L, de Vries H (2015). Comparing a video and text version of a Web-based computer-tailored intervention for obesity prevention: a randomized controlled trial. J Med Internet Res.

[74] Stanczyk N, Bolman C, van Adrichem M, Candel M, Muris J, de Vries H (2014). Comparison of text and video computer-tailored interventions for smoking cessation: randomized controlled trial. J Med Internet Res.

[75] Williams L, Ball K, Crawford D (2010). Why do some socioeconomically disadvantaged women eat better than others? An investigation of the personal, social and environmental correlates of fruit and vegetable consumption. Appetite.

[76] Janssen E, Sugiyama T, Winkler E, de Vries H, te Poel F, Owen N. Psychosocial correlates of leisure-time walking among Australian adults of lower and higher socio-economic status. Health Educ Res. 2010;25(2):316-324. 10.1093/her/cyp012 19307317

[77] Brug J, Steenhuis I, van Assema P, de Vries H (1996). The impact of a computer-tailored nutrition intervention. Prev Med.

[78] Sassen B, Kok G, Mesters I, Crutzen R, Cremers A, Vanhees L. A web-based intervention for health professionals and patients to decrease cardiovascular risk attributable to physical inactivity: development process. JMIR Res Protoc. 2012;1(2):e21–e21. 10.2196/resprot.1804 PMC3626153 23612470

[79] van Stralen MM, Kok G, de Vries H, Mudde AN, Bolman C, Lechner L. The Active plus protocol: systematic development of two theory- and evidence-based tailored physical activity interventions for the over-fifties. BMC Public Health. 2008;8:399–399. 10.1186/1471-2458-8-399 PMC2613403 19055806

[80] Lloyd JJ, Logan S, Greaves CJ, Wyatt KM. Evidence, theory and context--using intervention mapping to develop a school-based intervention to prevent obesity in children. Int J Behav Nutr Phys Act. 2011;8:73–73. 10.1186/1479-5868-8-73 PMC3152876 21752261

[81] Stevens GWJM, Kamperman AM, de Jong JTVM, Crijnen AAM (2005). Aanbevelingen voor de werving van allochtone respondenten voor onderzoek. TSG.

[82] Petty RE, Barden J, Wheeler SC, DiClemente RJ, Crosby RA, Kegler M (2002). The elaboration likelihood model pf persuasion: health promotions that yield sustained behavioral change. Emerging theories in health promotion practice and research.

[83] McNeill BW, Stoltenberg CD (1989). Reconceptualizing social influence in counseling: The Elaboration Likelihood Model. J Couns Psychol.

[84] Petty RE, Wegener DT, Fabrigar LR (1997). Attitudes and attitude change. Annu Rev Psychol.

[85] O’Keefe DJ (2002). Persuasion: theory and research.

[86] Gist ME, Mitchell TB (1992). Self-efficacy: a theoretical analysis of its determinants and malleability. Acad Manage Rev.

[87] Compton JA, Pfau MW (2005). Inoculation theory of resistance to influence at maturity: Recent progress in theory development and application and suggestions for future research. Communication yearbook.

[88] Heaney CA, Israel BA, Glanz K, Rimer BK, Viswanath K (2008). Social networks and social support. Health behavior and health education: Theory, research, and practice.

